# Association Between the Dietary Inflammatory Index and Life’s Essential 8 in Older Adults Based on Gut Microbiota Profiles

**DOI:** 10.3390/nu17193050

**Published:** 2025-09-24

**Authors:** Yuxiao Wu, Qianqian Chen, Rui Fan, Lixia Song, Shuyue Wang, Mei You, Meng Cai, Yong Li, Meihong Xu

**Affiliations:** 1 Department of Nutrition and Food Hygiene, School of Public Health, Peking University, Beijing 100191, China; 2 Beijing Key Laboratory of Toxicological Research and Risk Assessment for Food Safety, Peking University, Beijing 100191, China

**Keywords:** Dietary Inflammatory Index, Life’s Essential 8, gut microbiome, cardiovascular health

## Abstract

Background/Objectives: As the global population ages, cardiovascular disease (CVD) emerges as a critical challenge for public health, with chronic inflammation identified as a key contributing risk factor. As a modifiable lifestyle factor, diet plays a critical role in the prevention of CVD. Given the established link between diet and inflammation, clarifying the relationship between dietary inflammatory potential and cardiovascular health (CVH) is of significant public health importance. This study aimed to evaluate the association between dietary inflammatory potential and CVH in an elderly population, and to explore the related role of the gut microbiota. Methods: Dietary inflammatory potential was quantified using the Dietary Inflammatory Index (DII), CVH was assessed by the American Heart Association’s Life’s Essential 8 (LE8) score, and gut microbiome analysis was profiled by 16S rRNA gene sequencing. Results: Results showed that higher DII scores, indicative of a pro-inflammatory dietary pattern, were significantly linked to reduced LE8 scores, suggesting an inverse association between dietary inflammatory potential and CVH. Based on the gut microbiome, participants with high CVH exhibited greater α diversity compared with those with low CVH, while both α and β diversity were higher in the anti-inflammatory diet group than in the pro-inflammatory diet group. These results indicate that anti-inflammatory diets may be associated with better CVH, possibly through the preservation of the ecological balance of the gut microbiota. Correlation analyses further pointed to several genera potentially associated with both dietary inflammatory potential and CVH. Functional predictions suggested that variation in dietary inflammatory potential could be linked to differences in microbial metabolic functions relevant to energy, lipid and glucose metabolism, and inflammatory processes. Conclusions: In conclusion, this study provides novel evidence linking dietary inflammatory potential, gut microbiota, and CVH in older adults, and offers preliminary insights for dietary interventions and microbiota-targeted strategies in CVD prevention.

## 1. Introduction

Among older adults, cardiovascular disease (CVD) persists as the foremost contributor to global morbidity and mortality, creating a heavy strain on both healthcare delivery and public health [1]. To more accurately capture and quantify cardiovascular health (CVH) in the general population, the American Heart Association (AHA) recently introduced the Life’s Essential 8 (LE8) framework, an updated framework building upon the earlier Life’s Simple 7 (LS7) [2]. Compared with LS7, LE8 incorporates sleep health as a novel metric and revises all seven original components with updated definitions and scoring standards [2]. This expanded framework provides a more comprehensive and precise assessment of CVH, with robust evidence linking it to reduced risks of CVD, other chronic conditions, and mortality, thereby reinforcing its role as a reliable tool for CVD research [3,4,5,6].

Dietary intakes are key determinants in regulating chronic systemic inflammation, which is a well-established contributor to cardiovascular risk. The Dietary Inflammatory Index (DII) is a validated instrument designed to assess the inflammatory potential of an individual’s diet, drawing on its associations with biomarkers including C-reactive protein (CRP), interleukin-6 (IL-6), and tumor necrosis factor-alpha (TNF-α) [7]. The DII has been widely employed in multiple research studies to investigate associations between dietary patterns and diverse health outcomes [8]. Higher DII scores, representing a dietary pattern with higher inflammatory potential, have been shown in previous studies to be closely associated with CVD and a range of cardiometabolic risk factors, including hypertension, dyslipidemia, and impaired glucose homeostasis [9,10,11,12,13,14,15]. Nevertheless, the index has several inherent limitations that are difficult to avoid, including reliance on self-reported dietary data and incomplete coverage of inflammatory biomarkers [16].

The gut microbiota plays a pivotal role in modulating host cardiometabolic health, and accumulating evidence has linked gut dysbiosis to a range of CVD-related phenotypes, including atherosclerotic progression, blood pressure regulation, lipid metabolism disorders, obesity, and vascular inflammation [17]. Notably, individuals with CVD often display characteristic alterations in microbial composition and reduced microbial diversity, underscoring dysbiosis as a common feature of cardiovascular pathophysiology [18,19,20]. Beyond compositional changes, functional perturbations of the microbiome may underlie the mechanisms through which gut microbes influence CVH. For example, microbial fermentation of dietary fibers produces short-chain fatty acids (SCFAs), which modulate inflammation, regulate vascular tone, lower blood pressure, and contribute to cardiovascular processes such as cardiac repair and arterial compliance [21,22,23]. By contrast, microbial metabolism of choline and carnitine generates trimethylamine N-oxide (TMAO), a metaorganismal metabolite causally linked to atherosclerosis, thrombosis, and vascular inflammation [24,25]. Gut microbes also modify the bile acid pool, thereby influencing lipid and glucose metabolism via receptors such as FXR and TGR5 [26]. More recently, phenylacetylglutamine (PAG), a phenylalanine-derived metabolite identified through metabolomics studies, has been linked to major adverse cardiovascular events (MACE) independent of traditional risk factors, and shown to promote adverse cardiovascular phenotypes through adrenergic receptor signaling [27]. Together, these pathways provide strong mechanistic plausibility for the gut microbiota in linking diet to CVH.

Moreover, diet is a critical determinant of gut microbial composition and function [28]. A recent systematic review of ten studies, most of which were cross-sectional and conducted in varied populations, reported weak-to-moderate associations between the DII and the gut microbiome [29]. Although findings on microbial diversity were inconsistent, lower DII scores reflecting anti-inflammatory diets were more consistently linked to favorable microbial profiles [29]. As noted above, both diet and the gut microbiota are closely linked to CVH: the diet inflammatory potential may affect cardiovascular risk, while the gut microbiota and its metabolites also play important roles in cardiovascular pathophysiology. Nevertheless, the interactions among diet, the gut microbiota, and CVH are inherently complex [30]. To date, studies simultaneously addressing diet inflammatory potential, the gut microbiota, and CVH remain limited, and their interrelationships are not yet well defined. Given that aging is accompanied by substantial alterations in metabolism, immune regulation, and gut microbial composition that collectively increase cardiovascular vulnerability, older adults represent a particularly relevant population for such investigations [31,32]. Furthermore, age-related decline in organ function and reduced metabolic flexibility heighten susceptibility to dietary inflammation and its adverse health consequences, providing an important context in which to investigate the interplay between diet, the microbiota, and CVH.

This study aims to investigate the association between the DII and cardiovascular health assessed by LE8 in older adults, with particular attention to the contribution of the gut microbiota. Although previous studies have examined associations among diet, the gut microbiome, and cardiovascular health, to our knowledge the LE8 framework has not yet been applied to this context, particularly in older adults. By examining gut microbial features alongside dietary patterns and CVH, the study may help clarify biological pathways through which dietary inflammation influences cardiovascular risk. Furthermore, identifying microbial signatures associated with lower DII scores and favorable LE8 profiles could inform microbiota-based prevention strategies and support the development of precision nutrition approaches in aging populations.

## 2. Methods

### 2.1. Study Population

This cross-sectional analysis was based on the baseline survey of the TALENTs trial (Targeting Aging and Longevity with Exogenous Nucleotides), a 4-month, pragmatic, single-center randomized controlled trial. A total of 301 community-dwelling adults aged 60–70 years were included. Baseline demographic characteristics and chronic disease history were obtained using structured questionnaires, as described in the published protocol [33]. Fecal samples were collected during the baseline phase, immediately processed, and stored at −80 °C. After quality control, 290 samples met the inclusion criteria and were analyzed. A participant-flow diagram is provided in Supplementary Figure S1. All participants provided written informed consent prior to study entry. The study was approved by the Biomedical Ethics Committee of Peking University (IRB00001052-21114) and registered with ClinicalTrials.gov (NCT05243108).

### 2.2. Assessment of Dietary Intake

Dietary intake was evaluated using the photo-assisted dietary intake assessment (PAD) method, which has been validated in our previous studies for accuracy and feasibility across diverse populations [34]. Prior to data collection, participants underwent standardized training and received real-time professional support. For three consecutive days, they photographed every meal before and after consumption, covering both routine and additional foods. The images were reviewed to estimate portion sizes and nutrient composition. Mean intake across the three consecutive recorded days was subsequently calculated from these records.

### 2.3. Inflammatory Potential of the Diet

Dietary intake data, obtained from the three consecutive days of photo-assisted dietary intake assessment described above, were used to calculate the DII. The inflammatory potential of the diet was quantified using the DII as described by Shivappa et al. [7]. 22 dietary components were included in the calculation: energy, protein, carbohydrate, fat, cholesterol, saturated fatty acids, monounsaturated fatty acids, polyunsaturated fatty acids, dietary fiber, folate, vitamin A, vitamin B1, vitamin B2, vitamin B3, vitamin B6, vitamin B12, vitamin C, vitamin D, vitamin E, zinc, magnesium, iron, and selenium. Food-derived bioactive compounds, such as eugenol, saffron, isoflavones, chili pepper, and rosemary, were excluded due to unavailable intake data. In accordance with DII methodology, calculations were considered valid when fewer than 30 parameters were available [7,35].

For every dietary factor, a Z-score was calculated as the difference between the mean intake across the three consecutive recorded days and the global daily mean intake, divided by the global standard deviation. Z-scores were converted to percentile scores, multiplied by two, and subtracted from one to achieve a symmetrical distribution. Each percentile score was then multiplied by its corresponding inflammatory effect score to generate a component-specific DII value. The overall DII score for each participant was obtained by summing the values of all dietary components. Positive scores reflected a pro-inflammatory dietary pattern, while negative scores reflected an anti-inflammatory pattern. For this analysis, participants were categorized into tertiles according to their DII scores.

### 2.4. Measurement of CVH

CVH was measured with the LE8 score, defined by the AHA [2], encompassing four behavioral domains (diet, physical activity (PA), nicotine exposure, and sleep) and four biological factors (body mass index (BMI), blood lipids, glucose, and blood pressure (BP)). Dietary quality was evaluated with the Mediterranean Eating Pattern Assessment (MEPA) tool [36]. Data on PA, nicotine exposure, sleep habits, diabetes status, and medication use were collected through self-reported questionnaires. Standardized physical examinations provided anthropometric indices, including height, weight, and BP, from which BMI was derived as weight in kilograms divided by squared height in meters. Blood samples were analyzed for non-HDL cholesterol, fasting plasma glucose, and glycated hemoglobin A1c (HbA1c). Following the 2022 AHA guidelines, each LE8 metric was assigned a score ranging from 0 to 100, with the overall CVH score computed as the arithmetic mean of all components. Based on total LE8 values, participants were classified into high (80–100), moderate (50–79), or low (0–49) CVH groups. The detailed scoring algorithm and cut-off values for each component are presented in Supplementary Table S1.

### 2.5. Measurement of Skin Advanced Glycation End-Products (AGEs)

Skin AGEs were assessed non-invasively by skin autofluorescence (SAF) using a desktop device (AGE Reader™, DiagnOptics Technologies, Groningen, The Netherlands). Measurements were taken on the right forearm with participants in a seated position. For each participant, three consecutive readings were obtained, and the mean value was used for analysis. The AGE Reader quantifies AGEs based on their fluorescent properties and expresses results in arbitrary units (AU). This method has been clinically validated against skin biopsy, the gold standard for AGEs determination, in large-scale clinical trials, with reproducibility confirmed by negligible intra-individual and day-to-day variation [37].

### 2.6. 16S rRNA Gene Amplicon Sequencing

Microbial DNA was extracted from fecal samples using the MGIEasy Fecal Genomic DNA (Meta) Extraction Kit (BGI, Shenzhen, China). The V3–V4 hypervariable region of the bacterial 16S rRNA gene was amplified by polymerase chain reaction (PCR) with primers PF (5′-ACTCCTACGGGGAGGCAGCAG-3′) and PR (5′-GGACTACNNGGGGTATCTAAT-3′). Amplicons were purified with Agencourt AMPure XP magnetic beads (Beckman Coulter, UK), eluted in buffer, and prepared for library construction. Library fragment size and concentration were evaluated using an Agilent 2100 Bioanalyzer (Agilent, CA, USA). Libraries that passed quality control were sequenced on the MGISEQ-2000 platform (BGI, Shenzhen, China) to generate paired-end reads according to the insert length. Raw reads were filtered and merged using FLASH (v1.2.11) to generate tags from the V3–V4 region. Tags were clustered into operational taxonomic units (OTUs) at 97% sequence similarity using USEARCH (v7.0.1090), and chimeras were removed with UCHIME (v4.2.40). Representative sequences from each OTUs were taxonomically assigned by alignment against the Ribosomal Database Project (RDP; [38]) using the RDP Classifier (v2.2) with a confidence threshold of 0.6.

### 2.7. Bioinformatic Analysis

Alpha diversity at the operational taxonomic units (OTUs) level was evaluated in R (v4.5.1) using the Chao1, ACE, and Shannon indices. Beta diversity was calculated with unweighted and weighted UniFrac distances and group-level differences in microbial community structure were tested with Analysis of Similarities (ANOSIM). To further assess group separation based on microbial profiles, Partial Least Squares Discriminant Analysis (PLS-DA) was performed with the mixOmics package. PICRUSt2 (v2.3.0-b) was applied to predict microbial functional potential from 16S rRNA gene sequences. Overlaps of OTUs between groups were visualized as Venn diagrams generated with the VennDiagram package. Correlation heatmaps were produced using heatmaps to identify microbial taxa potentially associated with LE8 scores or dietary variables.

### 2.8. Statistical Analysis

Baseline characteristics were summarized as: mean ± standard deviation (SD) for normally distributed continuous variables, and frequency (percentage) for categorical variables. Group differences were assessed using ANOVA for normally distributed continuous data or the Kruskal–Wallis test for skewed data, and by chi-square or Fisher’s exact tests for categorical data where appropriate. Pearson correlation analysis was performed to examine the linear relationship between the DII and LE8. In addition, linear regression models were used to further assess the association between DII and LE8. Models were first unadjusted and then adjusted for age, sex and energy intake. Regression results are presented as β coefficients with 95% confidence intervals (CIs). R (v4.5.1) was used for all analyses, adopting a threshold of two-sided *p* < 0.05 for statistical significance.

## 3. Results

### 3.1. Demographic Characteristics by DII Groups

Demographic characteristics were compared among participants across the tertiles of DII (T1: LDII, T2: MDII, T3: HDII). Participants in the highest tertile of DII had significantly higher DII scores, reflecting a more pro-inflammatory dietary pattern, compared with those in the lowest tertile (*p*  <  0.001). No significant differences were observed among groups in age, sex distribution, or history of diabetes mellitus and CVD (Table 1).

### 3.2. Association Between DII and LE8

To investigate the relationship between the DII and LE8, differences in LE8 scores among the LDII, MDII, and HDII groups were evaluated (Table 2). The LE8 total score was significantly higher in the LDII group compared with the MDII and HDII groups (*p* = 0.009), indicating more favorable CVH with lower dietary inflammatory potential. Among individual LE8 components, the diet score showed marked differences across groups (*p* < 0.001), with the highest score in the LDII group and the lowest in the HDII group, reflecting substantial variation in diet quality. The PA score was lowest in the HDII group, although the difference did not reach statistical significance (*p* = 0.086). Other components, including smoking, sleep, BMI, blood lipids, blood glucose, and BP, showed no significant differences, but demonstrated trends consistent with the LE8 total score. According to LE8 classification, the distribution of low, moderate, and high CVH levels differed significantly among DII groups (*p* = 0.042). Furthermore, subcutaneous AGEs (Advanced Glycation End-products) levels increased progressively with higher DII, with significantly higher values in the HDII group compared with the LDII group (*p* = 0.029), suggesting a potential link between dietary inflammatory load and AGEs accumulation.

**Figure 1:**
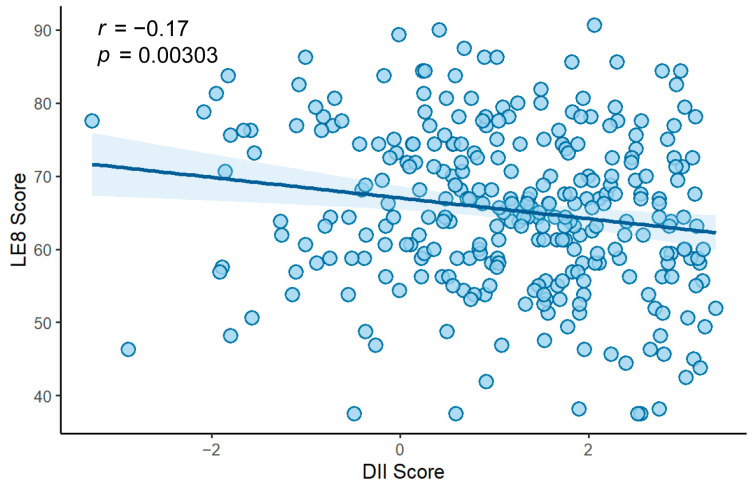
Scatter plot illustrating the association between Dietary Inflammatory Index (DII) and Life’s Essential 8 (LE8) score (*n* = 301).

Additional, Pearson correlation analysis demonstrated a significant negative association between DII and LE8 scores (*r* = −0.17, *p* = 0.030; Figure 1), indicating that higher dietary inflammatory potential was related to poorer CVH. To further validate this relationship, linear regression analyses were conducted. When modeled as a continuous variable, each one-point increase in DII score was associated with a 1.582-point decrease in the LE8 score after adjustment for age, sex and energy intake (adjusted *β* = −1.582, 95% CI: −2.679 to −0.484). This association remained significant in the unadjusted model (*β* = −1.415, 95% CI: −2.346 to −0.483), suggesting robustness of the finding. When DII was categorized into three levels using the LDII group as the reference, participants in the HDII group had significantly lower LE8 scores (adjusted *β* = −4.741, 95% CI: −8.124 to −1.358, adjusted for age, sex and energy intake), whereas the MDII group showed a nonsignificant trend toward lower scores (adjusted *β* = −2.121, 95% CI: −5.183 to 0.940). Taken together, these results consistently suggest that higher dietary inflammatory potential is associated with poorer CVH, both when DII is analyzed as a continuous measure and when categorized into groups.

### 3.3. Gut Microbiota Composition Across CVH Groups

**Figure 2:**
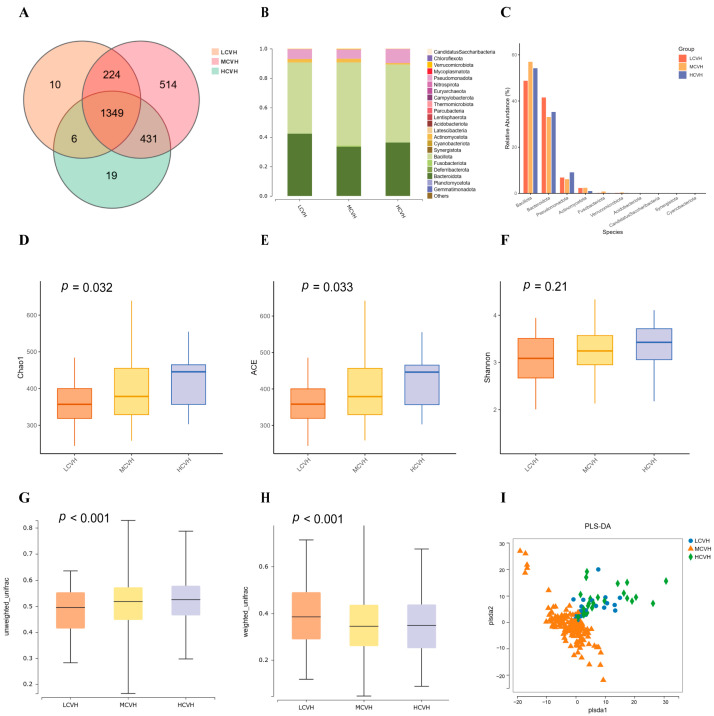
Distribution of identified intestinal flora across LE8 Cardiovascular Health (CVH) Groups (*n* = 290). (**A**) Venn diagram showing the distribution of Operational Taxonomic Units (OTUs) among the three LE8 CVH groups. (**B**) Composition of intestinal flora at the phylum level across the three groups. (**C**) Differences in relative abundance of top 10 flora species at the phylum level among the groups. Comparison of alpha diversity using the (**D**) Chao index, (**E**) ACE and (**F**) Shannon index among the groups. The beta diversity based on (**G**) unweighted, and (**H**) weighted UniFrac distances analysis among the groups. (**I**) Partial Least Squares Discriminant Analysis (PLS-DA) plot showing microbial community separation among the groups.

To explore the potential role of the gut microbiota in mediating the impact of the DII on CVH, we first compared the gut microbial composition among the three CVH groups: low CVH (LCVH), moderate CVH (MCVH), and high CVH (HCVH). A total of 1589, 2518, and 1805 OTUs were identified in the LCVH, MCVH, and HCVH groups, respectively, with 1349 OTUs shared across all three groups (Figure 2A). At the phylum level, the gut microbiota of all groups was dominated by *Bacillota*, *Bacteroidota*, *Pseudomonadota*, and *Verrucomicrobiota*, which together accounted for the majority of the community. Although the relative abundances of these dominant phyla showed variation among the groups, the differences were not statistically significant, but they indicate distinct overall community patterns (Figure 2B,C).

Alpha diversity was assessed using the Chao1, ACE, and Shannon indices (Figure 2D–F). Both Chao1 and ACE showed significant differences among groups (*p* < 0.05), while Shannon did not. All three indices, however, indicated a gradual increase in richness and diversity from the LCVH to the HCVH group, suggesting that higher CVH status is associated with a more diverse gut microbiota. Beta diversity based on UniFrac distances also revealed significant differences in microbial community structure across groups (Figure 2G,H). Unweighted UniFrac distances analysis, which emphasizes the presence or absence of taxa, showed greater dissimilarity in the HCVH group, reflecting differences in rare microbial members. Weighted UniFrac distances analysis, which incorporates relative abundances, highlighted larger differences in the LCVH group, pointing to distinct variation in dominant taxa. PLS-DA analysis showed a clear separation trend among the three CVH groups (Figure 2I). The distinction of the MCVH group from the others was more pronounced. This observation may be partly explained by the unequal sample size distribution across the groups, with the larger MCVH sample providing greater stability and tighter clustering.

### 3.4. Gut Microbiota Composition Across DII Groups

**Figure 3:**
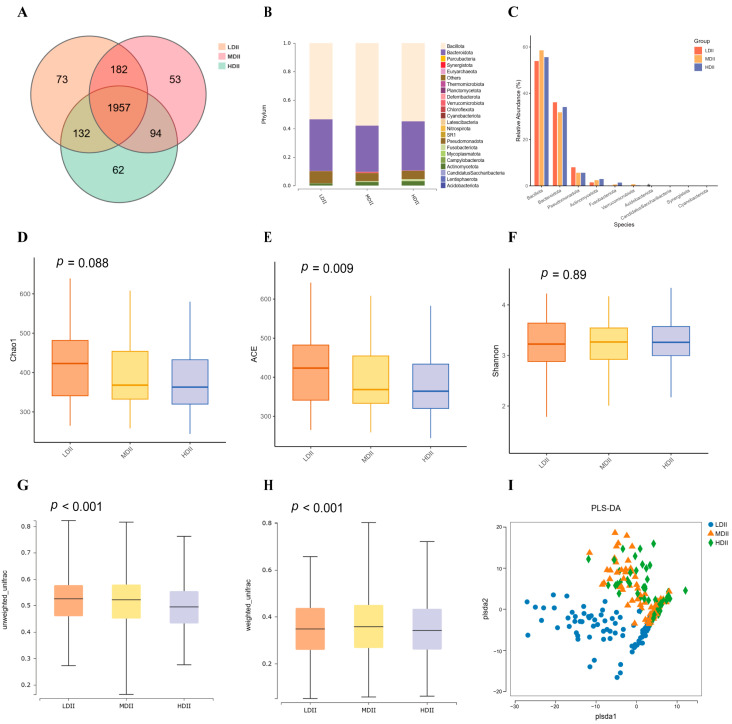
Distribution of identified intestinal flora across DII Groups (*n* = 290). (**A**) Venn diagram showing the distribution of Operational Taxonomic Units (OTUs) among the three LE8 CVH groups. (**B**) Composition of intestinal flora at the phylum level across the three groups. (**C**) Differences in relative abundance of top 10 flora species at the phylum level among the groups. Comparison of alpha diversity using the (**D**) Chao index, (**E**) ACE and (**F**) Shannon index among the groups. The beta diversity based on (**G**) unweighted, and (**H**) weighted UniFrac distances analysis among the groups. (**I**) Partial Least Squares Discriminant Analysis (PLS-DA) plot showing microbial community separation among the groups.

In addition, gut microbial composition was compared across the three DII groups. A total of 2344, 2286, and 2245 OTUs were identified in the LDII, MDII, and HDII groups, respectively, with 1957 OTUs shared among all groups (Figure 3A). At the phylum level, 21 bacterial phyla were detected, among which *Bacillota*, *Bacteroidota*, *Pseudomonadota*, and *Actinomycetota* were predominant (Figure 3B,C). The distribution of these dominant phyla varied across DII groups, and the relative abundance of *Acidobacteriota* was significantly higher in the LDII group than in the MDII and HDII groups (*p* < 0.05, Figure 3C).

Alpha diversity, assessed by the Chao1, ACE, and Shannon indices, showed that Chao1 and ACE differed significantly among groups (*p* < 0.05), whereas Shannon did not. All three indices indicated higher richness and diversity in the LDII group, followed by the MDII group, with the lowest levels observed in the HDII group (Figure 3D–F). Beta diversity based on both unweighted and weighted UniFrac distances revealed significant differences in microbial community structure (Figure 3G,H). In both analyses, the HDII group clustered separately with lower diversity, while the LDII and MDII groups showed similar community profiles. PLS-DA further supported the separation of microbial compositions across the three DII groups (Figure 3I).

### 3.5. Identification of Key Gut Microbial Genera Associated with Both DII and LE8

**Figure 4:**
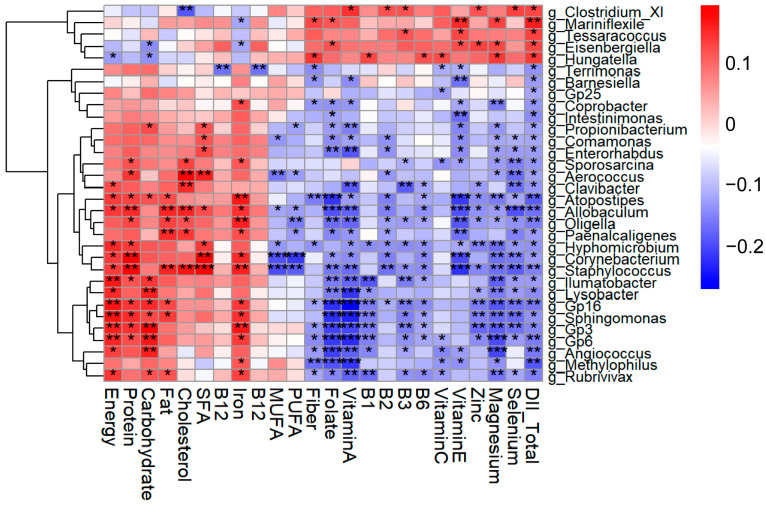
Clustered heatmap of Spearman correlations between the relative abundance of selected bacterial genera and the Dietary Inflammatory Index (DII) (*n* = 290). * *p* < 0.05; ** *p* < 0.01; *** *p* < 0.001.

Correlation analysis between the DII and genus-level relative abundances identified 32 candidate genera (Figure 4). Five genera—*Tessaracoccus*, *Mariniflexile*, *Hungatella*, *Eisenbergiella*, and *Clostridium*_*XI*—were positively correlated with DII, whereas the remaining genera showed negative correlations. Representative negatively correlated taxa included *Allobaculum*, *Atopostipes*, *Coprobacter*, *Barnesiella*, *Intestinimonas*. Interestingly, genera positively associated with pro-inflammatory nutrients—energy, protein, carbohydrate, fat, cholesterol, saturated fatty acids, vitamin B12, and iron —were generally inversely correlated with anti-inflammatory nutrients; conversely, genera positively related to anti-inflammatory nutrients tended to be negatively associated with these pro-inflammatory components. Collectively, these findings suggest that the DII-associated genera may reflect the inflammatory potential of the diet and could provide preliminary insights into potential microbiome markers of dietary inflammatory burden.

To further explore the microbial genera potentially mediating the relationship between DII and CVH, we examined the associations between LE8 scores and the relative abundance of the 32 DII-associated genera. As shown in Table 3, 6 genera were identified as key taxa related to both DII and LE8. Among them, *Eisenbergiella* was positively associated with DII but negatively associated with LE8. In contrast, the other 5 genera—such as *Allobaculum*, *Atopostipes*, and *Barnesiella*—showed negative associations with DII and positive associations with LE8 score.

These findings suggest that these genera may provide preliminary clues to the links between dietary inflammatory potential and CVH.

### 3.6. Functional Prediction of Key Gut Microbiota

**Figure 5:**
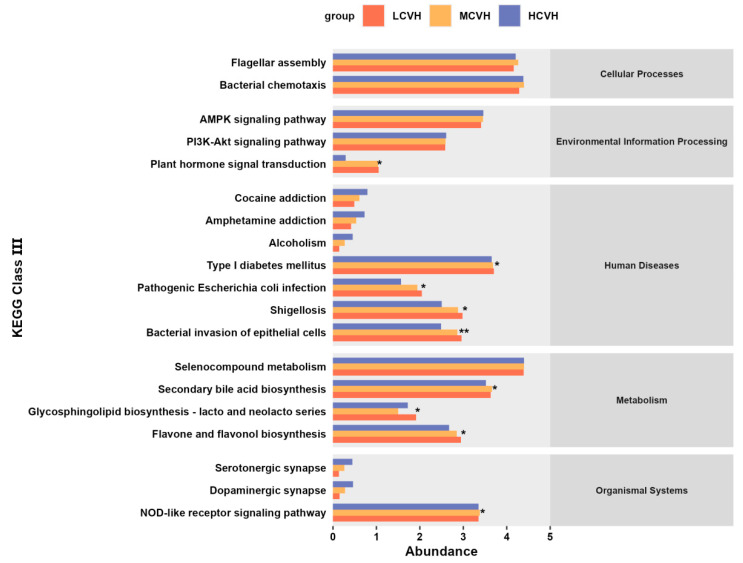
PICRUSt-based functional prediction of key gut microbial genera showing significantly enriched KEGG pathways (*n* = 290). * *p* < 0.05, ** *p* < 0.01, represent intergroup differences, based on Kruskal-Walli’s test.

To further investigate the possible pathways through which the selected gut microbial genera might be related to dietary inflammation and CVH, we employed PICRUSt to infer functional gene profiles and their enrichment in Kyoto Encyclopedia of Genes and Genomes (KEGG) pathways. As shown in Figure 5, several KEGG pathways were significantly enriched. Within the Cellular Processes and Environmental Information Processing categories, pathways such as flagellar assembly, bacterial chemotaxis, AMPK signaling, and PI3K–Akt signaling were enriched, with the plant hormone signal transduction pathway additionally showing significant intergroup differences (*p* < 0.05). In the Human Diseases category, enriched pathways included type I diabetes mellitus, pathogenic Escherichia coli infection, shigellosis, and bacterial invasion of epithelial cells, with the latter showing the strongest group differences (*p* < 0.01). In the Metabolism category, enriched pathways included selenocompound metabolism, secondary bile acid biosynthesis, glycosphingolipid biosynthesis (lacto and neolacto series), and flavone and flavonol biosynthesis, of which the latter two showed significant intergroup differences (both *p* < 0.05). In addition, the NOD-like receptor signaling pathway in the Organismal Systems category also displayed significant intergroup variation (*p* < 0.05). These findings suggest that the key microbial genera may affect CVH by engaging in significantly enriched pathways related to host energy metabolism, immune responses, and inflammation.

## 4. Discussion

Systemic chronic inflammation constitutes a significant risk factor as well as an underlying pathogenic mechanism for CVD [39]. As a modifiable lifestyle factor, dietary intake represents an important strategy for preventing cardiovascular events [40,41]. As a validated measure derived from the literature, the DII quantifies the overall inflammatory capacity of dietary intake and has shown consistent associations with systemic inflammation markers [7]. Therefore, given the modifiable nature of diet and its established link to inflammation, elucidating the relationship between dietary inflammatory potential and CVH holds substantial public health significance. In this cross-sectional study, we systematically examined, for the first time, the association between the DII and the LE8 scores, a comprehensive metric of CVH proposed by the AHA. The results revealed a significant inverse correlation between DII and LE8 scores, indicating that higher DII scores (reflecting a more pro-inflammatory diet) are associated with lower LE8 scores (indicating poorer CVH). These findings suggest that pro-inflammatory dietary patterns are associated with poorer CVH, whereas adopting dietary patterns with anti-inflammatory potential may be linked to more favorable cardiovascular outcomes.

The gut-heart axis represents a dynamic, bidirectional network in which gut health, particularly the homeostasis of the gut microbiota, plays a critical role in maintaining the host’s immune and metabolic balance [42,43,44]. Emerging evidence has established this axis as a central component of CVH, influencing its regulation both directly and indirectly, and may be critically involved in the development and progression of CVD [45,46,47]. Dysbiosis of the gut microbiota, characterized by reduced microbial richness and diversity coupled with the expansion of specific pathogenic bacteria, has been associated with the development and progression of various diseases, including CVD [43]. Among the numerous host-endogenous and host-exogenous factors involved, diet emerges as a critical determinant of the structural and functional composition of the gut microbial community [48]. Previous studies have shown that a high-DII diet can disrupt gut microbiota homeostasis [49,50]. Consistent with these findings, our study revealed that individuals consuming a pro-inflammatory diet (HDII) were associated with differences in gut microbiota composition compared with those consuming an anti-inflammatory diet (LDII). Moreover, we observed markedly reduced alpha and beta diversity in the HDII group. These results further support existing evidence that DII scores are associated with microbial diversity and richness, as well as with overall shifts in microbiome structure [29]. Additionally, it has been reported that patients with CVD exhibit reduced gut microbial gene richness and diversity compared with healthy controls [21,51,52,53]. Our findings are consistent with these observations, showing that participants with poorer CVH had differences in gut microbial community structure and lower alpha diversity.

In this study, correlation analysis identified several genera that were potentially associated with both the DII and LE8 scores. Among these, *Eisenbergiella* was found to be positively correlated with pro-inflammatory dietary patterns and negatively correlated with CVH status, suggesting that this genus may act as a detrimental taxon in the context of CVH. This finding aligns with prior reports indicating that *Eisenbergiella* may function as a pro-inflammatory genus and is positively associated with elevated CRP levels [54]. Additionally, *Eisenbergiella* belongs to the family *Lachnospiraceae*. An increased abundance of *Lachnospiraceae* has been associated with the development of metabolic diseases, including diabetes [55]. Conversely, several taxa—including *Allobaculum*, *Atopostipes*, *Barnesiella*, *Coprobacter*, and *Intestinimonas*, among others—have been associated with potential cardiovascular benefits, while pro-inflammatory diets have been shown to diminish their abundance. The physiological roles of these genera in relation to host health are increasingly being elucidated through contemporary microbiome research. *Allobaculum* is associated with improved intestinal health and demonstrates the capacity to produce SCFAs [56]. It also exhibits anti-inflammatory properties, supports metabolic function, and may contribute to reducing insulin resistance [57,58,59]. Research suggests that *Atopostipes* may exert beneficial effects on host metabolism, as evidenced by its negative correlation with triglyceride levels and its potential protective role in high-fat diet-induced obesity [60,61,62]. *Barnesiella* contributes to multiple protective processes, including bile acid metabolism, the promotion of glucose homeostasis and insulin sensitivity, and interactions with immune cells that maintain an anti-inflammatory intestinal environment [63,64,65]. Furthermore, a decreased abundance of *Barnesiella* has been associated with aggravated cerebral small vessel disease, suggesting that it may also support neurovascular health [66]. According to studies, *Coprobacter* has been linked to lowering total serum cholesterol, alleviating ulcerative colitis, promoting energy metabolism, and enhancing intestinal barrier function through anti-inflammatory activity [67,68,69]. Similarly, *Intestinimonas*, as a butyrate-producing bacterium, helps improve symptoms of type 2 diabetes and contributes to metabolic regulation [70,71]. Together, these beneficial microbes contribute to metabolic health, enhance gut barrier function, and help maintain systemic immune and inflammatory homeostasis via anti-inflammatory effects and metabolite production.

Growing research points to the gut microbiota may influence CVH through multiple mechanistic pathways. Intestinal microorganisms contribute to the biosynthesis of a wide range of bioactive metabolites that help sustain normal human physiological functions. Among these, microbial-derived metabolites such as SCFAs, bile acids, TMAO, tryptophan, and indole derivatives have been closely linked to cardiovascular outcomes [72]. Dietary intake of choline, lecithin, and L-carnitine leads to their microbial conversion into trimethylamine (TMA), which is subsequently oxidized in the liver to TMAO—a metabolite strongly associated with atherosclerosis, thrombogenesis, and vascular inflammation [73]. In contrast, circulating SCFAs—including acetate, propionate, and butyrate—which are largely produced by gut bacteria, have demonstrated protective roles in heart failure prevention, BP regulation, and the maintenance of gut barrier integrity via enhanced mucus production and anti-inflammatory actions [74,75]. A bidirectional interaction exists between bile acids and the gut microbiota: while bile acids exert antimicrobial effects that shape microbial composition, gut bacteria modify primary bile acids into secondary species through processes such as deconjugation and dehydroxylation. These secondary bile acids function as signaling mediators that influence host physiology by activating nuclear receptors [76,77]. Disturbance of this equilibrium has been associated with the development of cardiometabolic disorders [78]. Furthermore, a weakened intestinal barrier also promotes the leakage of bacterial-derived substances, for instance lipopolysaccharide (LPS), into circulation. LPS can initiate a pro-inflammatory state through the activation of Toll-like receptor (TLR) signaling pathways [17,79]. Elevated circulating LPS levels have been significantly correlated with an increased risk of MACE, underscoring the possible role of gut-derived inflammation in cardiovascular pathology [80]. Collectively, these findings suggest that pro-inflammatory dietary patterns may contribute to induce gut microbiota dysbiosis, characterized by a reduction in SCFA-producing taxa and an expansion of LPS-producing bacteria, thereby potentially compromising intestinal barrier function, elevating TMAO levels, and promoting systemic inflammation. Conversely, anti-inflammatory diets may help foster beneficial microbial communities, enhance SCFAs production, reinforce epithelial barrier integrity, and suppress inflammatory pathways, potentially contributing to cardiovascular protection. Nevertheless, the precise mechanisms through which the gut microbiota may be linked to CVH remain complex and these mechanistic inferences are speculative and require further validation in longitudinal and experimental studies.

To explore potential mechanisms linking dietary inflammatory potential, the gut microbiota, and CVH, we performed a functional prediction analysis on the identified key microbial genera. As these are derived from 16S rRNA gene-based functional inference, the results represent indirect proxies rather than direct measurements of microbial metabolites or pathways and should be interpreted with caution. The results indicate that the PI3K/Akt and AMPK signaling pathways may represent central pathways potentially involved. AMPK serves as a central cellular energy and metabolic sensor, playing a pivotal regulatory role in maintaining cardiometabolic homeostasis and protecting against CVD. Its activation exerts cardiovascular protective effects through multiple mechanisms, including improvements in glucose and lipid metabolism, reductions in blood pressure, suppression of reactive oxygen species generation, and enhancement of nitric oxide (NO) bioavailability [81]. Notably, gut microbiota-derived SCFAs, such as butyrate and propionate, have been shown to activate AMPK, thereby modulating mitochondrial function and cellular energy homeostasis [82]. Furthermore, AMPK activation is involved in the regulation of glucose metabolism, underscoring its importance for cardiometabolic health [83]. The PI3K/Akt pathway also functions as a critical regulator of cellular metabolism and survival. Evidence suggests that SCFAs may activate PI3K/Akt signaling, thereby ameliorating insulin resistance and promoting normal glucose homeostasis [84,85,86]. Importantly, there is close crosstalk between the AMPK and PI3K/Akt pathways. Activated AMPK not only directly phosphorylates eNOS at Ser1177 to enhance its activity but also amplifies eNOS activation via the upstream PI3K/Akt pathway, thereby synergistically promoting NO production, attenuating inflammation, and ultimately mitigating atherosclerotic progression [87]. Thus, our findings indicate that dietary inflammatory potential is related to alterations in gut microbial ecology and metabolic functions—such as bile acid and SCFA production—which may, in turn, be associated with host energy metabolism, glucose–lipid metabolism, and inflammatory status, and with cardiometabolic and cardiovascular outcomes. The potential mechanisms are speculative and may involve both AMPK and PI3K/Akt signaling pathways; however, the precise modes of action remain to be elucidated and require further mechanistic and experimental validation.

This study is the first to investigate the association between CVH levels, as assessed by the LE8 score, and dietary inflammatory potential, measured by the DII, in an elderly population, and to preliminarily explore the potential role of the gut microbiome in this relationship—a topic that has received limited attention in previous research. However, several limitations should be acknowledged. Firstly, the sample size was relatively small due to practical constraints, and future studies with larger cohorts will be required to validate these findings. Secondly, the study population was derived from a single geographic cohort, which may limit the generalizability of the results to other populations with different ethnic, cultural, or environmental backgrounds. Thirdly, as a cross-sectional study, the present results cannot establish causal relationships, nor can they provide phased diagnostic or interventional recommendations such as those derived from clinical trials. In addition, the possibility of reverse causality cannot be excluded, as poorer CVH status may itself influence dietary patterns or gut microbiota composition. Fourthly, although a PAD method was used to assess dietary intake, this approach may still be subject to measurement error and reporting bias. Fifthly, although 16S rRNA gene sequencing was applied to characterize the gut microbiota, this method has inherent limitations, particularly its restricted taxonomic and functional resolution. The functional predictions generated by PICRUSt represent only indirect inferences rather than direct mechanistic evidence. In future investigations, metagenomic sequencing could provide higher-resolution insights into microbial functions. Furthermore, the lack of targeted metabolomic data further constrains mechanistic interpretation of the microbiota’s role. Therefore, integrating metagenomics with metabolomics will be essential for more robust validation. In addition, while correlation analyses identified several genera associated with DII and LE8 at the *p*-value level, none of these associations remained significant after FDR correction. As such, these results should be regarded as exploratory and hypothesis-generating, requiring cautious interpretation and validation in larger, independent cohorts, and future studies incorporating formal mediation analyses together with metabolomic profiling will be needed to rigorously examine potential causal pathways. Finally, although adjustments were made for important covariates, unmeasured or unknown factors may still have influenced the associations observed in this study, and residual confounding cannot be completely excluded. Diet represents an important modifiable behavioral factor with significant implications for the maintenance of CVH and the prevention of related diseases. Future research should move beyond cross-sectional associations through prospective cohorts to establish temporal relationships, controlled feeding studies to test how dietary modifications in DII influence microbial composition and metabolite profiles, and randomized trials of microbiota-targeted interventions in older adults with high DII to provide evidence for precision nutritional strategies in the prevention and management of CVD.

## 5. Conclusions

In summary, this study provides initial evidence of a possible association between dietary inflammatory potential and CVH, with the gut microbiota potentially involved in this association, together with the suggestion of several genera that may contribute to these effects. These findings provide preliminary insights into the “diet–microbiota–host” interplay in cardiometabolic health and indicate that anti-inflammatory dietary patterns could be explored further as a potential approach for CVD prevention and management, pending confirmation in longitudinal and mechanistic studies.

## Figures and Tables

**Table 1:** Demographic Characteristics by DII Groups ^a^ (*n* = 301).

Characteristics	LDII (*n* = 101)	MDII (*n* = 101)	HDII (*n* = 99)	*p*-Value ^b^
DII score	−0.32 ± 0.86	1.30 ± 0.37	2.56 ± 0.43	<0.001 ***
Age	65.58 ± 2.75	65.70 ± 2.59	65.70 ± 2.70	0.939
Male	33 (32.7%)	29 (28.7%)	31 (31.3%)	0.826
History of diabetes mellitus	6 (5.9%)	15 (14.9%)	8 (8.1%)	0.082
History of cardiovascular disease	14 (13.9%)	23 (22.8%)	26 (26.3%)	0.084

^a^ Values are presented as mean  ±  standard deviation (SD) for normally distributed continuous variables, and frequency (percentage) for categorical variables. ^b^ ANOVA for continuous variables and Chi-squared test for categorical variables. *** *p* < 0.001.

**Table 2:** Association between Dietary Inflammation Index (DII) and Cardiovascular Health (CVH) Indicators ^a^ (*n* = 301).

CVH Indicators	LDII (*n* = 101)	MDII (*n* = 101)	HDII (*n* = 99)	*p*-Value ^b^
LE8 score	67.61 ± 11.08	65.54 ± 9.57	62.89 ± 11.76	0.009 **
Diet score	37.97 ± 13.81	32.18 ± 11.37	26.77 ± 6.44	<0.001 ***
PA score	45.15 ± 41.99	41.58 ± 38.72	32.83 ± 40.00	0.086
Smoke score	88.37 ± 29.49	91.09 ± 26.36	85.86 ± 32.17	0.454
Sleep score	81.88 ± 24.73	82.08 ± 25.66	81.21 ± 28.79	0.971
BMI score	83.86 ± 21.43	85.79 ± 18.90	83.23 ± 22.58	0.668
Blood Lipids score	66.14 ± 29.09	63.37 ± 27.29	64.04 ± 28.85	0.770
Blood Glucose score	79.50 ± 22.02	76.93 ± 24.57	76.57 ± 25.40	0.640
BP score	57.97 ± 31.44	51.29 ± 31.22	52.63 ± 29.30	0.264
CVH level				0.042 *
Low CVH	7 (6.9%)	4 (4.0%)	14 (14.1%)	
Moderate CVH	81 (80.2%)	89 (88.1%)	79 (79.8%)	
High CVH	13 (12.9%)	8 (7.9%)	6 (6.1%)	
Skin AGEs	2.34 ± 0.38	2.42 ± 0.45	2.52 ± 0.56	0.029 *

PA: physical activity; BMI: body mass index; BP: blood pressure; CVH: cardiovascular health; AGEs: Advanced Glycation End-products. ^a^ Values are presented as mean  ±  standard deviation (SD) for normally distributed continuous variables, and frequency (percentage) for categorical variables. ^b^ ANOVA for continuous variables and Chi-squared test for categorical variables. * *p* < 0.05; ** *p* < 0.01; *** *p* < 0.001.

**Table 3:** Correlations of the relative abundance of the screened genera with DII and LE8 (*n* = 290).

Gut Microbiota at the Genus Level		DII Score	LE8 Score	LE8-Diet	LE8-PA	LE8-Smoke	LE8-Sleep	LE8-BMI	LE8-BloodLipids	LE8-BloodGlucose	LE8-BP
*g_Allobaculum*	ρ	−0.167	0.033	0.026	0.01	0.069	−0.033	−0.014	0.049	0.024	0.016
	*p*	0.004 **	0.573	0.655	0.871	0.242	0.581	0.817	0.408	0.683	0.785
*g_Atopostipes*	ρ	−0.182	0.058	−0.014	0.043	0.095	−0.007	0.036	0.033	−0.013	0.004
	*p*	0.002 **	0.327	0.818	0.468	0.106	0.906	0.546	0.578	0.830	0.949
*g_Barnesiella*	ρ	−0.13	0.03	0.031	−0.036	0.026	−0.012	0.019	0.029	0.04	0.066
	*p*	0.027 *	0.612	0.597	0.546	0.656	0.839	0.752	0.622	0.499	0.265
*g_Coprobacter*	ρ	−0.133	0.078	0.036	0.03	−0.011	−0.014	−0.026	0.005	0.108	0.128
	*p*	0.023 *	0.187	0.538	0.616	0.856	0.810	0.657	0.939	0.066	0.029 *
*g_Eisenbergiella*	ρ	0.129	−0.051	−0.059	−0.109	0.014	−0.029	0.158	0.014	−0.036	0.002
	*p*	0.028 *	0.390	0.319	0.064	0.814	0.625	0.007 **	0.819	0.546	0.978
*g_Intestinimonas*	ρ	−0.143	0.087	0.02	−0.023	0.092	0.034	0.105	0.088	0.029	0.042
	*p*	0.015 *	0.141	0.731	0.701	0.119	0.559	0.074	0.133	0.626	0.481

PA: physical activity; BMI: body mass index; BP: blood pressure. * *p* < 0.05; ** *p* < 0.01.

## Data Availability

The original contributions presented in the study are included in the article/Supplementary Material. Due to specific legal restrictions, the data are not publicly available, and further inquiries can be directed to the corresponding author.

## Supplementary Materials

The following supporting information can be downloaded at: https://www.mdpi.com/article/10.3390/nu17193050/s1, Figure S1: Study flow; Figure S2: Scatter plot of DII and LE8; Table S1: Definition and scoring of LE8; Table S2: Association between DII and LE8 scores from linear regression models; Table S3: Comparison of each dietary component of DII scores; Tables S4–S6: Results of sensitivity analyses.

## Abbreviations

CVD	cardiovascular disease
CVH	cardiovascular health
AHA	American Heart Association
LE8	Life’s Essential 8
PA	physical activity
BMI	body mass index
BP	blood pressure
DII	Dietary Inflammatory Index
CRP	C-reactive protein
IL-6	interleukin-6
TNF-α	tumor necrosis factor-alpha
PAD	photo-assisted dietary intake assessment
MEPA	Mediterranean Eating Pattern Assessment
HbA1c	glycated hemoglobin A1c
OTUs	operational taxonomic units
ANOSIM	Analysis of Similarities
PLS-DA	Partial Least Squares Discriminant Analysis
CI	confidence intervals
AGEs	Advanced Glycation End-products
KEGG	Kyoto Encyclopedia of Genes and Genomes
SCFAs	short-chain fatty acids
TMAO	trimethylamine N-oxide
TMA	trimethylamine
LPS	lipopolysaccharide
TLR	Toll-like receptor
MACE	major adverse cardiovascular events
NO	nitric oxide
