# Supplementary material for: Association Between the Dietary Inflammatory Index and Life’s Essential 8 in Older Adults Based on Gut Microbiota Profiles
Source: Nutrients. 2025 Sep 24;17(19):3050. doi: 10.3390/nu17193050 (PMC12525535; doi:10.3390/nu17193050)
Supplement: Supplementary file 1 [file nutrients-17-03050-s001.zip › nutrients-3863037-supplementary.pdf]

Supplementary materials

List of Supplementary Figures

Supplementary Figure S1. Overall flow of the study. ·· 3

Supplementary Figure S2. Scatter plot illustrating the association between Dietary Inflammatory Index (DII) and Life's Essential 8 (LE8) score (sensitivity analysis, n = 290).································ 5

List of Supplementary Tables

Supplementary Table S1. Definition and scoring approach for the American Heart Association's Life's Essential 8 score.·· 1

Supplementary Table S2. Association between Dietary Inflammatory Index (DII) and Life's Essential 8 (LE8) scores from linear regression models (n = 301).··· 2

Supplementary Table S3. Comparison of each dietary component of DII scores by DII Groupsa (n = 301).·· 2

Supplementary Table S4. Demographic characteristics by DII groups (sensitivity analysis, n = 290). ·· 4

Supplementary Table S5. Association between Dietary Inflammatory Index (DII) and cardiovascular health (CVH) indicators (sensitivity analysis, n = 290). ·· 4

Supplementary Table S6. Association between Dietary Inflammatory Index (DII) and Life's Essential 8 (LE8) scores from linear regression models (sensitivity analysis, n = 290). ······················ 5

Supplementary Table S1. Definition and scoring approach for the American Heart Association's Life's Essential 8 score[1].

Domain	CVH metric	Method of measurement	Quantification of CVH metric
Health behaviors	Diet	Daily dietary intake following the MEPA eating pattern	Scores of MEPA eating pattern adherence<br>**Scoring:**<br><u>Points</u> <u>MEPA score (points)</u><br>100 15–16<br>80 12–14<br>50 8–11<br>25 4–7<br>0 0–3
	Physical activity	Measurement: Self-reported minutes of moderate or vigorous PA per week	Metric: Minutes of moderate- (or greater) intensity activity per week:<br>**Scoring:**<br><u>Points</u> <u>Minutes</u><br>100 ≥150<br>90 120–149<br>80 90–119<br>60 60–89<br>40 30–59<br>20 1–29<br>0 0
	Nicotine exposure	Measurement: Self-reported use of cigarettes or inhaled nicotine delivery system (NDS)	Metric: Combustible tobacco use or inhaled NDS use; or secondhand smoke exposure<br>**Scoring:**<br><u>Points</u> <u>Status</u><br>100 Never smoker<br>75 Former smoker, quit ≥5 y<br>50 Former smoker, quit 1–<5 y<br>25 Former smoker, quit <1 y, or currently using inhaled NDS<br>0 Current smoker<br>Subtract 20 points (unless score is 0) for living with active indoor smoker in home
	Sleep health	Measurement: Self-reported average hours of sleep per night	Metric: Average hours of sleep per night<br>**Scoring:**<br><u>Point</u> <u>Level</u><br>100 7–<9<br>90 9–<10<br>70 6–<7<br>40 5–<6 or ≥10<br>20 4–<5<br>0 <4
Health factors	BMI	Measurement: Body weight (kilograms) divided by height squared (meters squared)	Metric: BMI (kg/m^2)<br>**Scoring:**<br><u>Points</u> <u>Level</u><br>100 <25<br>70 25.0–29.9<br>30 30.0–34.9<br>15 35.0–39.9<br>0 ≥40.0
	Blood lipids	Measurement: Plasma total and HDL cholesterol with calculation of non–HDL cholesterol	Metric: Non–HDL cholesterol (mg/dL)<br>**Scoring:**<br><u>Points</u> <u>Level</u><br>100 <130<br>60 130–159<br>40 160–189<br>20 190–219<br>0 ≥220<br>If drug-treated level, subtract 20 points

	Blood glucose	Measurement: Fasting blood glucose (FBG) or casual HbA1c	Metric: FBG (mg/dL) or HbA1c (%)<br>**Scoring:**<br>Points Level<br>100 No history of diabetes and FBG <100 (or HbA1c <5.7)<br>60 No diabetes and FBG 100–125 (or HbA1c 5.7–6.4) (prediabetes)<br>40 Diabetes with HbA1c <7.0<br>30 Diabetes with HbA1c 7.0–7.9<br>20 Diabetes with HbA1c 8.0–8.9<br>10 Diabetes with Hb A1c 9.0–9.9<br>0 Diabetes with HbA1c ≥10.0
	Blood pressure	Measurement: Appropriately measured systolic and diastolic blood pressure	Metric: Systolic and diastolic BPs (mm Hg)<br>**Scoring:**<br>Points Level<br>100 <120/<80 (optimal)<br>75 120–129/<80 (elevated)<br>50 130–139 or 80–89 (stage 1 hypertension)<br>25 140–159 or 90–99<br>0 ≥160 or ≥100<br>Subtract 20 points if treated level

Supplementary Table S2. Association between Dietary Inflammatory Index (DII) and Life's Essential 8 (LE8) scores from linear regression models (n = 301).

DII	LE8 score			
	β (95%CI) [a]	*P*-value	*β* (95%CI) [b]	*P*-value
DII as continuous variable	-1.415 (-2.346 to -0.483)	0.003 **	-1.582 (-2.679 to -0.484)	0.0049 **
DII as categorical variable				
LDII	Reference		Reference	
MDII	-2.067 (-5.067 to 0.934)	0.1763	-2.121 (-5.183 to 0.940)	0.1737
HDII	-4.714 (-7.729 to -1.698)	0.0023 **	-4.741 (-8.124 to -1.358)	0.0062 **

[a] Model 1: Unadjusted linear regression.

[b] Model 2: Adjusted for age, sex and energy intake.

* P <0.05**; P <0.01; *** P <0.001.

β values represent regression coefficients with 95% confidence intervals (CIs).

Supplementary Table S3. Comparison of each dietary component of DII scores by DII Groups[a] (n = 301).

Diertary scores	LDII (n = 101)	MDII (n = 101)	HDII (n = 99)	*P*-value
Energy score	-0.05 ± 0.14	-0.11 ± 0.11	-0.15 ± 0.06	<0.001 ***
Protein score	0.00 ± 0.02	-0.01 ± 0.01	-0.02 ± 0.01	<0.001 ***
Carbohydrate score	-0.05 ± 0.06	-0.08 ± 0.04	-0.09 ± 0.04	<0.001 ***
Total fat score	-0.04 ± 0.24	-0.09 ± 0.23	-0.18 ± 0.19	0.001 **
Cholesterol score	0.08 ± 0.06	0.07 ± 0.07	0.06 ± 0.08	0.0185 *
SFA score	-0.33 ± 0.12	-0.33 ± 0.13	-0.35 ± 0.10	0.5452
MUFA score	0.01 ± 0.00	0.01 ± 0.00	0.01 ± 0.00	0.365
PUFA score	0.23 ± 0.19	0.30 ± 0.08	0.33 ± 0.02	<0.001 ***

Fibre score	0.43 ± 0.35	0.64 ± 0.05	0.66 ± 0.01	<0.001 ***
Folic acid score	-0.15 ± 0.07	-0.05 ± 0.13	0.10 ± 0.12	<0.001 ***
Vitamin A score	0.12 ± 0.22	0.23 ± 0.13	0.32 ± 0.08	<0.001 ***
Thiamin score	0.03 ± 0.05	0.05 ± 0.04	0.07 ± 0.03	<0.001 ***
Riboflavin score	0.03 ± 0.03	0.04 ± 0.02	0.05 ± 0.02	<0.001 ***
Vitamin B3 score	0.11 ± 0.10	0.17 ± 0.06	0.21 ± 0.03	<0.001 ***
Vitamin B6 score	-0.28 ± 0.12	-0.17 ± 0.14	0.03 ± 0.21	<0.001 ***
Vitamin B12 score	0.10 ± 0.03	0.10 ± 0.02	0.10 ± 0.04	0.5896
Vitamin C score	-0.24 ± 0.27	0.02 ± 0.28	0.31 ± 0.19	<0.001 ***
Vitamin E score	-0.27 ± 0.24	0.08 ± 0.34	0.39 ± 0.08	<0.001 ***
Zn score	-0.06 ± 0.23	0.12 ± 0.21	0.25 ± 0.12	<0.001 ***
Mg score	-0.07 ± 0.20	0.18 ± 0.13	0.34 ± 0.09	<0.001 ***
Fe score	0.02 ± 0.02	-0.01 ± 0.02	-0.02 ± 0.01	<0.001 ***
Se score	0.05 ± 0.11	0.11 ± 0.08	0.15 ± 0.04	<0.001 ***

SFA: Saturated fat; MUFA: Monounsaturated fat; PUFA: Polyunsaturated fat.

[a]Values are presented as mean ± standard deviation (SD).

[b] ANOVA for continuous variables.

* P <0.05; ** P <0.01; *** P <0.001.

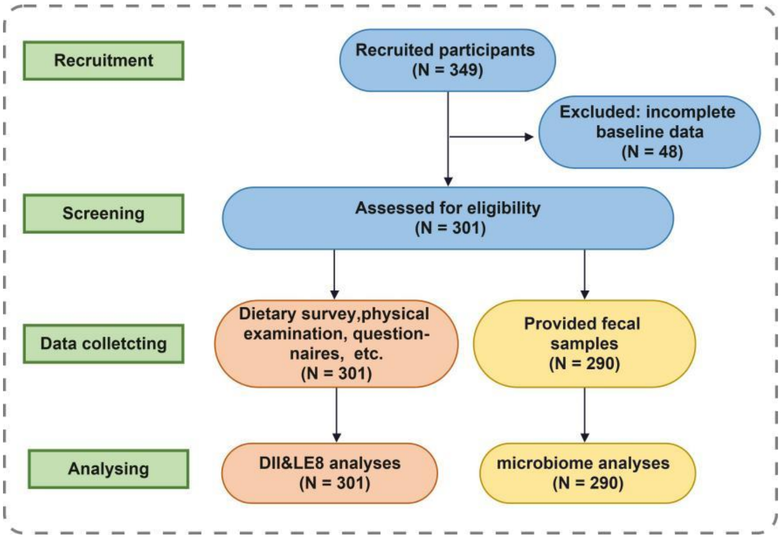

Supplementary Figure S1. Overall flow of the study.

To assess the robustness of our findings, we conducted sensitivity analyses restricted to participants with available fecal samples (n = 290). The corresponding results are presented in Supplementary Table S4–S6 and Supplementary Figure S2. These analyses yielded results that were consistent with those from the full analytic sample (n = 301), supporting the stability of our main conclusions.

Supplementary Table S4. Demographic characteristics by DII groups (sensitivity analysis, n = 290)[a].

Characteristics	LDII (n = 99)	MDII (n = 99)	HDII (n = 92)	*P*-value [b]
DII score	-0.33 ± 0.86	1.30 ± 0.37	2.57 ± 0.43	< 0.001 ***
Age	65.60 ± 2.72	65.67 ± 2.60	65.64 ± 2.71	0.9825
Male	32 (32.3%)	27 (27.3%)	28 (30.4%)	0.7359
History of diabetes mellitus	6 (6.1%)	15 (15.2%)	7 (7.6%)	0.0694
History of cardiovascular disease	14 (14.1%)	23 (23.2%)	24 (26.1%)	0.1036

[a] Values are presented as mean ± standard deviation (SD) for normally distributed continuous variables, and frequency (percentage) for categorical variables.

[b] ANOVA for continuous variables and Chi-squared test for categorical variables.

*** *P* <0.001.

Supplementary Table S5. Association between Dietary Inflammatory Index (DII) and cardiovascular health (CVH) indicators (sensitivity analysis, n = 290)[a].

CVH Indicators	LDII (n = 99)	MDII (n = 99)	HDII (n = 92)	*P*-value[b]
LE8 score	67.68 ± 10.97	65.49 ± 9.61	62.65 ± 11.81	0.0062 **
Diet score	37.98 ± 13.83	32.07 ± 11.32	26.90 ± 6.66	< 0.001 ***
PA score	45.05 ± 41.81	40.61 ± 38.49	30.65 ± 39.11	0.0406 *
Smoke score	89.14 ± 28.38	91.41 ± 26.28	85.87 ± 31.95	0.4141
Sleep score	82.12 ± 24.92	82.02 ± 25.83	80.22 ± 29.50	0.8599
BMI score	83.54 ± 21.53	85.81 ± 18.98	82.72 ± 22.34	0.5696
Blood Lipids score	66.46 ± 29.25	64.04 ± 26.80	65.22 ± 28.88	0.8342
Blood Glucose score	80.00 ± 21.00	76.87 ± 24.65	76.52 ± 25.78	0.534
BP score	57.12 ± 31.18	51.06 ± 31.45	53.10 ± 28.62	0.3649
CVH level				0.0184 *
Low CVH	6 (6.1%)	4 (4.0%)	14 (15.2%)	
Moderate CVH	80 (80.8%)	87 (87.9%)	73 (79.3%)	
High CVH	13 (13.1%)	8 (8.1%)	5 (5.4%)	
Skin AGEs	2.31 ± 0.29	2.41 ± 0.44	2.48 ± 0.54	0.0322 *

PA: physical activity; BMI: body mass index; BP: blood pressure; CVH: cardiovascular health; AGEs: Advanced Glycation End-products.

[a] Values are presented as mean ± standard deviation (SD) for normally distributed continuous variables, and frequency (percentage) for categorical variables.

[b] ANOVA for continuous variables and Chi-squared test for categorical variables.

* *P* <0.05; ** *P* <0.01; *** *P* <0.001.

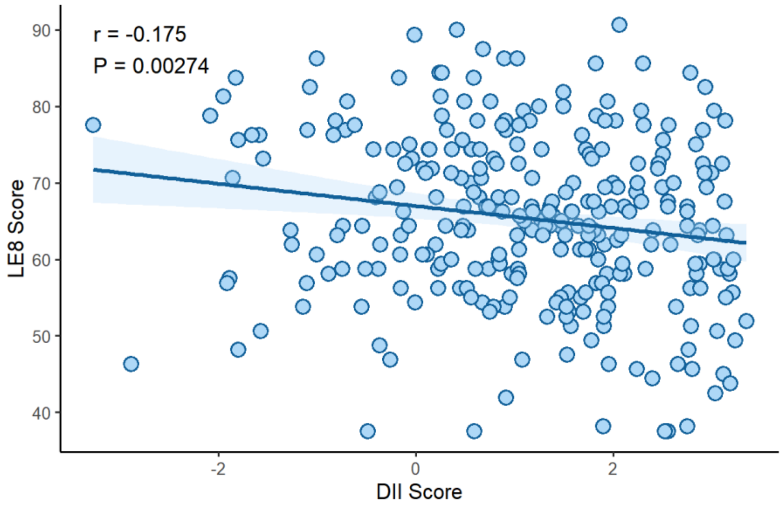

Supplementary Figure S2. Scatter plot illustrating the association between Dietary Inflammatory Index (DII) and Life's Essential 8 (LE8) score (sensitivity analysis, n = 290).

Supplementary Table S6. Association between Dietary Inflammatory Index (DII) and Life's Essential 8 (LE8) scores from linear regression models (sensitivity analysis, n = 290).

DII	LE8 score			
	β (95%CI) [a]	*P*-value	β (95%CI) [b]	*P*-value
DII as continuous variable	-1.415 (-2.396 to -0.506)	0.0027 **	-1.646 (-2.760 to -0.532)	0.0039 **
DII as categorical variable				
LDII	Reference		Reference	
MDII	-2.191 (-5.214 to 0.833)	0.1549	-2.327 (-5.411 to 0.758)	0.1388
HDII	-5.027 (-8.107 to -1.947)	0.0015 **	-5.184 (-8.658 to -1.710)	0.0036 **

[a] Model 1: Unadjusted linear regression.

[b] Model 2: Adjusted for age, sex and energy intake.

* P <0.05**; P <0.01; *** P <0.001.

β values represent regression coefficients with 95% confidence intervals (CIs).

References

1. Lloyd-Jones DM, Allen NB, Anderson CAM, Black T, Brewer LC, Foraker RE, Grandner MA, Lavretsky H, Perak AM, Sharma G, Rosamond W; American Heart Association. Life's Essential 8: Updating and Enhancing the American Heart Association's Construct of Cardiovascular Health: A Presidential Advisory From the American Heart Association. Circulation. 2022 Aug 2;146(5):e18-e43.
